# Social distancing, population density, and spread of COVID-19 in England: a longitudinal study

**DOI:** 10.3399/bjgpopen20X101116

**Published:** 2020-07-08

**Authors:** Peter Tammes

**Affiliations:** 1 Population Health Sciences, Bristol Medical School, University of Bristol, Bristol, UK

**Keywords:** COVID-19, incidence rate, population density, primary health care, social distancing, England, SARS-CoV-2

## Abstract

**Background:**

The UK government introduced social distancing measures between 16–22 March 2020, aiming to slow down transmission of COVID-19.

**Aim:**

To explore the spreading of COVID-19 in relation to population density after the introduction of social distancing measures.

**Design & setting:**

Longitudinal design with 5-weekly COVID-19 incidence rates per 100 000 people for 149 English Upper Tier Local Authorities (UTLAs), between 16 March and 19 April 2020.

**Method:**

Multivariable multilevel model to analyse weekly incidence rates per 100 000 people; time was level-1 unit and UTLA level-2 unit. Population density was divided into quartiles. The model included an interaction between week and population density. Potential confounders were percentage aged ≥65, percentage non-white British, and percentage in two highest classes of the National Statistics Socioeconomic Classification. Co-variates were male life expectancy at birth, and COVID-19 prevalence rate per 100 000 people on March 15. Confounders and co-variates were standardised around the mean.

**Results:**

Incidence rates per 100 000 people peaked in the week of March 30–April 5, showing higher adjusted incidence rate per 100 000 people (46.2; 95% confidence interval [CI] = 40.6 to 51.8) in most densely populated ULTAs (quartile 4) than in less densely populated ULTAs (quartile 1: 33.3, 95% CI = 27.4 to 37.2; quartile 2: 35.9, 95% CI = 31.6 to 40.1). Thereafter, incidence rate dropped in the most densely populated ULTAs resulting in rate of 22.4 (95% CI = 16.9 to 28.0) in the week of April 13–19; this was lower than in quartiles 1, 2, and 3, respectively 31.4 (95% CI = 26.5 to 36.3), 34.2 (95% CI = 29.9 to 38.5), and 43.2 (95% CI = 39.0 to 47.4).

**Conclusion:**

After the introduction of social distancing measures, the incidence rates per 100 000 people dropped stronger in most densely populated ULTAs.

## Introduction

In England, the number of coronavirus disease 2019 (COVID-19) cases rose quickly from the beginning of March 2020.^1^ Between 16–22 March, the UK government introduced social distancing measures with the aim to slow down the transmission of the virus.^2^ Schools, restaurants, pubs, clubs, and indoor sport and leisure facilities had to close. People had to stay at home and were only allowed to travel for food and health reasons, during which they had to keep a 2-metre distance from each other where possible. Pregnant women, people aged ≥70 years, and those with certain health conditions were urged to self-isolate. This study’s aim is to explore the spreading of COVID-19 in relation to population density after the introduction of social distancing measures, as adherence to these measures might be most challenging in areas of high population density given the high levels of interpersonal contact.^3^

## Method

Public Health England (PHE) Dashboard provides daily cumulative counts of lab-confirmed COVID-19 cases for each of the 149 UTLAs; that is, the highest (unitary) tier of local government: London and metropolitan boroughs, and unitary districts in shire counties. Data were downloaded at the beginning of May, with the notion that only data from 5 days or more ago can be considered complete.^4^ This study used lab-confirmed cases for 16 March–19 April. Adding UTLAs’ population sizes^5^ to this datafile allowed the calculation of weekly regional incidence rates per 100 000 people.

To explore the spreading of COVID-19 in relation to population density, this study included potential confounding population characteristics: a) older age, as older people are said to be more susceptibility to COVID-19^6^ and are more likely to live in less densely populated areas;^7^ b) ethnicity, as ethnic minorities are overrepresented among COVID-19 cases^8,9^ and are more likely to live in more densely populated areas;^7^ c) higher occupational classes, as these people may have more flexible work arrangements, allowing them to better cope with the restrictions,^10^ and are more likely to live in cities.^7^ Population characteristics included as co-variates are d) healthier populations, and e) the COVID-19 prevalence rate per 100 000 people on March 15.

The Office for National Statistics^3^ provides relevant data for each of the 149 UTLAs: percentage aged ≥65 years, percentage non-white British, percentage in two highest classes of the National Statistics Socioeconomic Classification, male life expectancy at birth, and number of people per square kilometre (see Supplementary Table S1 for descriptive statistics). Linking these data to the weekly regional COVID-19 incidence rates per 100 000 people allowed the association of population density with changes in weekly regional COVID-19 incidence rates per 100 000 people.

A multilevel model was used to analyse repeated measurements^11^ for the following weeks: March 16–22, March 23–29, March 30-April 5, April 6–12, and April 13–19. Time was the level-1 unit and UTLA the level-2 unit, resulting in 745 observations in the model. The multivariable model included an interaction between weeks and population density. Population density was divided into quartiles: quartile 1 was the least densely populated UTLAs, and quartile 4 the most densely populated ULTAs. The potential confounders and covariates were standardised around the mean.

## Results

**Figure 1.**
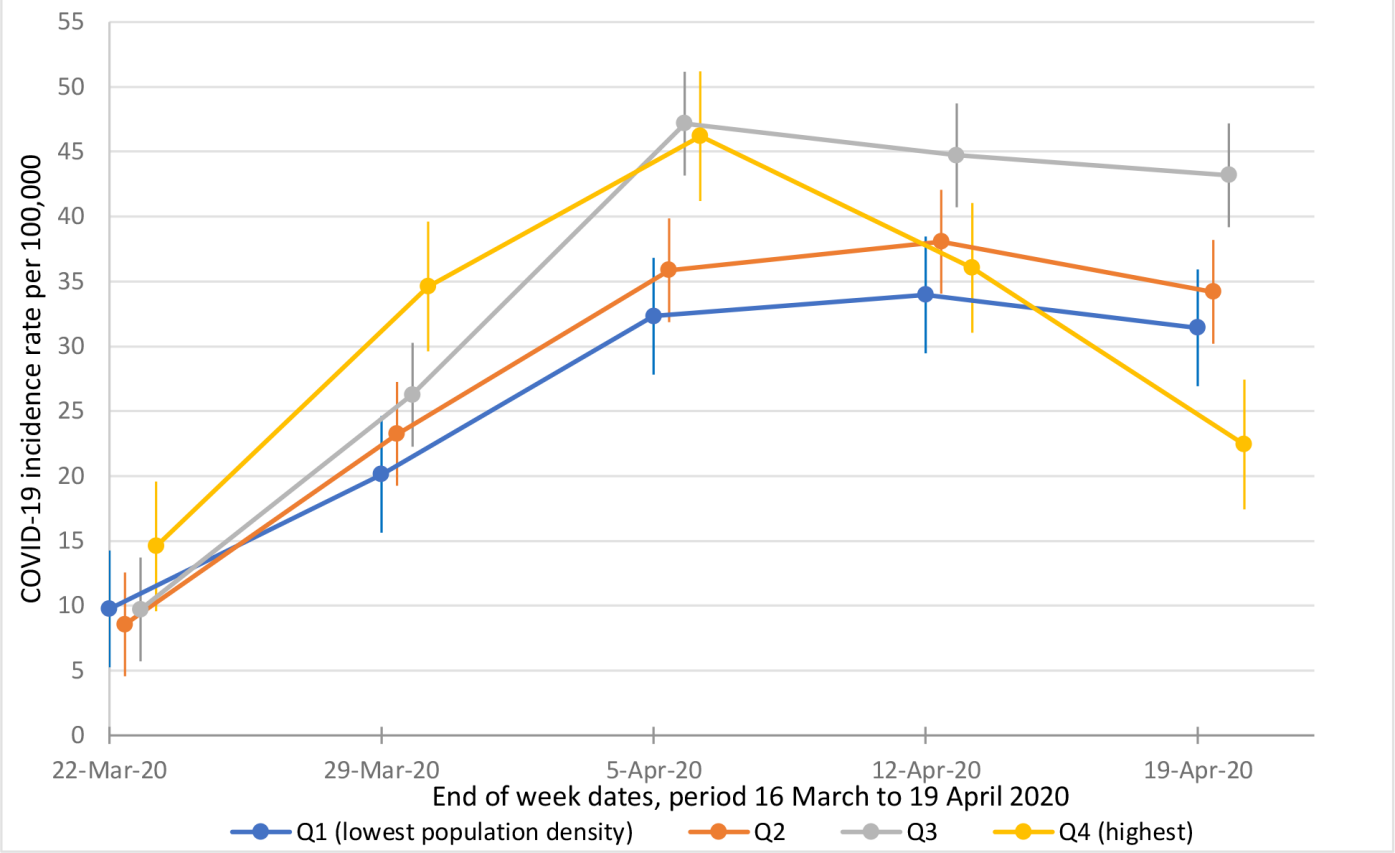
Estimates of B-coefficients (95% CI) from multilevel regression model for the association between population density and weekly regional COVID-19 incidence rates per 100 000 people, 16 March– 19 April 2020 (*n* = 745). Note: regression adjusted for percentage of population non-white British, percentage of population in two highest classes of the National Statistics Socioeconomic Classification, male life expectancy at birth, percentage aged ≥65 years, and COVID-19 prevalence rate per 100 000 people on March 15 ; covariates were standardised around the mean. 95% CI = 95% confidence interval; Q1= quartile 1 (lowest population density); Q4= quartile 4 (highest population density).

Adjusted incidence rates per 100 000 people for population density quartiles over time are presented in Figure 1. Weekly COVID-19 incidence rates per 100 000 people were highest in areas with higher population density (that is, quartiles 3 and 4), in the week of March 23–29, respectively 26.3 (95% CI = 22.1 to 30.5) and 34.6 (95% CI = 29.0 to 40.2), and in the week of March 30–April 5, respectively 47.2 (95% CI = 43.0 to 51.4) and 46.2 (95% CI = 40.6 to 51.8). Thereafter, incidence rate per 100 000 people dropped in the most densely populated ULTAs, resulting in a rate of 22.4 (95% CI = 16.9 to 28.0) in the week of April 13–19. This incidence rate is lower than for quartiles 1, 2, and 3, respectively 31.4 (95% CI = 26.5 to 36.3), 34.2 (95% CI = 29.9 to 38.5), and 43.2 (95% CI = 39.0 to 47.4).

## Discussion

### Summary

The UK government’s social distancing measures seem to have had the biggest impact in areas where people are living closest together. The weekly incidence rates per 100 000 people for the most densely populated UTLAs dropped from being the highest to being the lowest 4 weeks after social distancing measures were introduced.

### Strengths and limitations

The UK government’s central policy prioritised specific people to be tested.^12^ As a result, the lab-confirmed cases on the PHE Dashboard are probably an underestimation of the true number of COVID-19 cases, but testing practices should have been the same everywhere in England. Since this testing policy changed at the end of April, this longitudinal study used available data until April 20 to avoid measurement bias in the calculation of weekly COVID-19 incidence rates.

The lab-confirmed cases on the PHE Dashboard are patients who had probably been infected about a week before their test, as the median incubation period is 5–6 days.^13^ Given the time-lag between being infected and tested, the calculated incidence rates per 100 000 people might therefore rather be an indication of the spreading of the virus in the previous week. This may strengthen the conclusion that social distancing resulted in the strongest decrease in incidence rate in most densely populated areas. Although adherence to these distancing rules seems high,^14^ it is unknown whether there is a relationship between adherence and population density.

The study’s longitudinal approach allowed the comparison of incidence rates over time, and the association of population density with changes in incidence rates. However, given the ecological nature of the data (aggregated to UTLA-level), one cannot infer associations for individual patients.

### Comparison with existing literature

Some studies concluded that high population density catalyses the spread of COVID-19 as it increases contact rates.^3,8^ Avoiding contact is more difficult in higher population density areas or locations. Limiting contact in these areas seems, therefore, to be vital to slowing down the spread of COVID-19, as this study’s results show.

A study in the US noticed an increase of COVID-19 cases within areas that included a major airport,^15^ suggesting travel hubs are important sources of spreading. Studies on the US and UK concluded that introducing social distancing regulations reduced contact rate and physical proximity to others, resulting in reductions in COVID-19 incidence rates.^14,16^ Since areas with high population density tend to have or be close to major airports or travel hubs, regulations restricting mobility might have resulted in a stronger reduction in incidence rate within these areas.

### Implications for research and practice

Flaxman *et al* concluded that social distancing measures had an impact on reducing the transmission of COVID-19.^17^ This study showed that incidence rates strongly decreased in most densely populated areas. Others warned that easing social distancing restrictions should be considered carefully, as small increases in contact rates are likely to risk resurgence.^18^ This is a relevant warning given the higher contact rates in densely populated areas. More research is needed to understand why areas of high population density benefitted most from social distancing measures. This may inform policy to ease social distancing restrictions safely.

As long as such a policy is unclear, it is worthwhile continuing to follow guidelines on remote GP consultations.^19^ However, a delay in returning to ‘usual’ face-to-face primary care consultations will have further adverse consequences such as reduced presentation of patients with cancer symptoms.^20^ Introduction of additional measures may be necessary to ensure early diagnosis^20^ and that appropriate chronic disease management (such as for asthma)^21^ are not continuing to be compromised.
